# Recruitment Source and Participant Retention in a Digital HIV Prevention Intervention for Young Black and Latino Men and Transgender Women: Secondary Analysis of the HealthMpowerment 2.0 Randomized Controlled Trial

**DOI:** 10.2196/75793

**Published:** 2026-08-26

**Authors:** Willey Y Lin, Seul Ki Choi, Sabina Hirshfield, Marta I Mulawa, Dovie L Watson, Lisa B Hightow-Weidman, Kathryn E Muessig, José A Bauermeister

**Affiliations:** 1 Department of Family and Community Health, School of Nursing, University of Pennsylvania, 418 Curie Blvd, Rm 237L, Philadelphia, PA, 19104, United States, 1 6693339925; 2 Department of Medicine, SUNY Downstate Health Sciences University, Brooklyn, NY, United States; 3 School of Nursing, Duke University, Durham, NC, United States; 4 Department of Medicine, Perelman School of Medicine, University of Pennsylvania, Philadelphia, PA, United States; 5 Institute on Digital Health and Innovation, College of Nursing, Florida State University, Tallahassee, FL, United States

**Keywords:** HIV, men who have sex with men, mHealth, Hispanic Americans, African Americans, recruitment

## Abstract

**Background:**

Young Black and Latino men who have sex with men and transgender women who have sex with men (YBLMT) experience disproportionate HIV-related health disparities in the United States. Digital health interventions offer scalable HIV prevention and support services for these populations. However, recruitment strategies may influence both sample demographics and participant retention, which is critical for intervention effectiveness.

**Objective:**

This study examined whether recruitment source was associated with retention in a mobile health randomized controlled trial and assessed demographic differences across recruitment platforms.

**Methods:**

Data were drawn from the HealthMpowerment (HMP) 2.0 randomized controlled trial (N=750; July 2020 to September 2022). Participants aged 15 to 29 years were recruited through 4 sources: social media advertisements (n=202), partner-seeking apps (n=394), prior study contacts (n=118), and other sources (eg, community outreach, peer referrals; n=36). Retention was defined as completing at least 3 of 4 follow-up surveys over 12 months, equivalent to completing at least 80% (n=4) of all 5 study measurement occasions, consistent with the United States Preventive Services Task Force (USPSTF) criteria for cohort study follow-up. Chi-square and Fisher exact tests assessed unadjusted retention differences across recruitment sources. Multivariable logistic regression adjusting for age, HIV status, race, ethnicity, and gender identity estimated adjusted odds ratios (aOR) for the association between recruitment source and retention. A sensitivity analysis requiring completion of all 4 follow-up surveys was also conducted. A predictive logistic regression model incorporating Synthetic Minority Oversampling Technique (SMOTE) to address class imbalance further explored demographic and recruitment predictors of retention.

**Results:**

Recruitment source was significantly associated with retention (*χ*²_3_=36.62; *P*<.001). Participants recruited via social media (184/202, 91.1%) and prior study contacts (103/118, 87.3%) had higher retention than those recruited via partner-seeking apps (288/394, 73.1%) and other sources (23/36, 63.9%). After adjusting for demographics, social media (aOR 2.80, 95% CI 1.51‐5.18) and study contacts (aOR 2.11, 95% CI 1.14‐3.92) remained significantly associated with higher retention compared to partner-seeking apps. HIV-positive status (aOR 0.64) and gender-diverse identity (aOR 0.38) were independently associated with lower retention. The sensitivity analysis yielded directionally consistent findings, with the social media advantage remaining significant. The SMOTE-adjusted model improved recall for low-retention participants from 6% (2/33) to 64% (21/33) but reduced overall accuracy from 77.3% (116/150) to 64.7% (97/150).

**Conclusions:**

Recruitment source was associated with both participant characteristics and long-term retention in this digital HIV prevention intervention. Social media and prior research networks yielded higher retention, whereas partner-seeking apps reached populations at higher HIV risk but were associated with lower retention. Targeted retention strategies that account for both recruitment platform and participant characteristics (including HIV status and gender identity) may help optimize engagement and sample diversity in future digital health interventions targeting YBLMT.

## Introduction

Young Black and Latino men who have sex with men and transgender women who have sex with men (YBLMT) in the United States face disproportionate health disparities, including elevated rates of HIV infection and barriers to care [1]. These disparities are driven by intersectional stigmas related to race, ethnicity, sexuality, and HIV status, further compounded by geographic and logistical barriers such as living in rural or homophobic communities or being located far from research institutions [2]. As our day-to-day lives become increasingly digital, online health interventions offer opportunities to address these challenges by providing better privacy, confidentiality, and accessibility, thereby overcoming many geographical and logistical limitations [3,4].

While online health interventions enhance accessibility, recruitment strategies must also evolve to effectively reach underrepresented populations. Traditional methods, such as venue-based sampling and community outreach, often struggle to engage YBLMT due to concerns about selection bias, confidentiality, stigma, and logistical constraints [5]. For example, venue-based recruitment at LGBTQ+ events may exclude individuals who do not openly identify or participate in these spaces.

Online recruitment methods, including advertising on social media and dating apps, address some of these limitations by offering greater reach and privacy [3,4]. However, they also introduce new challenges, such as the risk of fraudulent responses, competition for ad visibility, and increasing recruitment costs [6,7]. Further, targeted digital advertising can drive up costs due to competition with commercial advertisers, particularly when targeting niche populations [6]. Balancing cost-effectiveness with strategies that ensure data integrity and minimize selection biases is essential for optimizing online recruitment efforts.

Beyond cost and accessibility, another critical consideration is how different recruitment sources influence the demographic composition of study samples and participant retention over time. Prior research suggests that recruitment sources not only shape who enrolls in a study but also impact how likely participants are to complete follow-up assessments [8]. For example, social media ads tend to attract younger, more tech-savvy participants who may exhibit higher retention [8], while dating apps may reach older and more diverse populations, including individuals at higher risk for HIV [8]. These differences influence sample diversity and retention, both of which are crucial for ensuring generalizable findings. While previous studies have explored how recruitment sources affect study engagement, limited research has systematically examined their impact on participant retention, particularly in digital health interventions targeting YBLMT.

Given the potential impact of recruitment strategies on participant diversity and retention, a more systematic understanding of these patterns is necessary for designing effective recruitment strategies. Identifying predictors of retention, such as demographic or recruitment factors linked to sustained participation, can help refine recruitment strategies to improve study retention. While recruitment source itself is not a causal determinant of retention, different platforms may attract participants with varying likelihoods of completing study follow-ups [8]. Understanding these recruitment-driven variations can help researchers optimize outreach to improve retention or adjust analyses to account for selection bias when comparing recruitment effectiveness.

Longitudinal data from the HealthMpowerment (HMP) 2.0 study provided an opportunity to explore predictors of study retention. HMP 2.0 is a mobile health intervention evaluated through a randomized controlled trial (RCT) designed to reduce intersectional stigma and improve HIV-related outcomes among YBLMT. The study used multiple recruitment sources, including paid ads on social media and partner-seeking apps, email listservs of past study contacts, and traditional community-based methods.

The objective of this study was to examine whether recruitment source was associated with participant retention in the HMP 2.0 RCT. We additionally explored demographic differences across recruitment sources and assessed demographic and recruitment predictors of retention. Ultimately, these insights aim to enhance inclusivity, optimize retention in online health interventions, and ensure that recruitment strategies are cost-effective and tailored to the needs of diverse populations.

## Methods

### Study Procedures

All data were collected as part of the 3-arm RCT evaluating HMP 2.0. The 3 arms were the tailored information-only control (attention control), researcher-created HMP network (intervention arm 1), and peer-referral network (intervention arm 2). Details of the original study design and intervention are described in the published protocol [9].

Participants were recruited between July 2020 and September 2022 through 4 primary sources: paid ads on social media platforms (eg, Facebook, Instagram, X [formerly Twitter]); paid ads on partner-seeking apps (eg, Jack’d, Scruff, Grindr); study contacts (ie, individuals who had previously participated in research conducted by the study team or affiliated projects and had consented to be recontacted about future studies through institutional email listservs); and other sources that included in-person recruitment, peer referrals, community outreach, and postings on LGBTQ+ forums. Ads on social media platforms and partner-seeking apps appeared in users’ feeds, inboxes, or app interfaces and directed interested individuals to an online Qualtrics screener to verify eligibility.

To be eligible for the study, individuals had to be between the ages of 15 and 29 years (inclusive) at the time of screening, identify as Black or African American and/or Latino or Hispanic (except participants referred by those in Intervention arm 2), reside in the United States, and speak and read English. They were also required to have regular access to a smartphone and either report anal intercourse in the past 6 months or have been recruited from a partner-seeking app. The study included individuals assigned male sex at birth, with no restrictions on their current gender identity.

Interested individuals completed an online screening survey [10], and those who met all study eligibility criteria were emailed a unique link to the study’s informed consent form. Upon providing consent, participants completed a 30 to 50-minute baseline computer-assisted self-interview (CASI) survey.

To maintain data integrity, a thorough fraud detection process was implemented that involved reviewing IP addresses, geolocation data, and comparing responses between the screening and baseline surveys. Additionally, participant data were cross-checked with the study’s participant database to detect duplicate or fraudulent entries. This process effectively identified and removed duplicate or fraudulent entries.

Participants who passed the validation checks were randomized into 1 of the 3 study arms using a computer-generated blocked randomization procedure stratified by HIV status. They were then provided a link to download the study app from their respective app store (ie, Apple’s App Store or Google Play). To support retention across the 12-month study period, participants received regular reminders via email, text messages, and in-app notifications. These efforts aimed to maximize completion rates for follow-up surveys conducted at 3, 6, 9, and 12 months.

Participants could receive up to US $490 in Amazon gift card incentives for completing study surveys and other study-related activities. The breakdown of incentives includes up to US $280 for completing the study surveys, up to US $140 for completing study test kits, up to US $20 for successfully referring participants to the study (intervention arm 2 only), and US $50 for completing an in-depth interview, if selected.

### Measures

#### Recruitment Sources

The primary independent variable was recruitment source, categorized as social media, partner-seeking apps, study contacts, and other sources. Each participant’s recruitment source was recorded at intake. Participants recruited via social media platforms (eg, Facebook, Instagram) and partner-seeking apps (eg, Jack’d, Scruff, Grindr) were classified according to the platform on which the study ad appeared. Platforms with fewer than 10 participants were grouped into their respective broad category rather than analyzed as stand-alone groups, as small cell sizes would have precluded meaningful statistical comparisons. Other sources comprised community-based recruitment methods including in-person outreach, peer referrals, and postings on LGBTQ+ forums.

#### Retention Metrics

Participant retention was defined as the completion of follow-up surveys at 3, 6, 9, and 12 months. For this analysis, retention was dichotomized into 2 groups: high retention (completion of at least 3 of 4 follow-up surveys) and low retention (completion of fewer than 3 surveys). Because all 750 enrolled participants completed the baseline assessment, this threshold equates to completing at least 4 (80%) of 5 total study measurement occasions, consistent with the United States Preventive Services Task Force (USPSTF) Quality Rating Criteria defining “Good” quality follow-up for cohort studies as greater than 80% retention [11].

Because there was an imbalance in retention categories, with high-retention participants significantly outnumbering low-retention participants, the Synthetic Minority Oversampling Technique (SMOTE) [12] was applied to adjust for class imbalance during model development. SMOTE was applied only to the training dataset, while the testing dataset retained its original class distribution to ensure unbiased evaluation of model performance.

#### Demographic Characteristics

Demographic characteristics were assessed using structured self-report items in the screening and baseline surveys. To ensure statistical robustness and consistency in analysis, these variables were categorized accordingly. Age was grouped into 3 categories: 15 to 19, 20 to 24, and 25 to 29 years. Ethnicity was classified as Hispanic or non-Hispanic. For race, because the survey allowed participants to select multiple racial identities, a binary categorization was used: participants who selected only “White” were categorized as “White,” while all others, including those who selected multiple racial categories, were grouped as “Non-White or Multiracial.” This approach was chosen to avoid misclassifying multiracial participants and is consistent with analytic approaches used in studies with similar survey instruments. Hispanic ethnicity was retained as a separate variable, as Latino identity is an ethnic rather than a racial category and captures a distinct dimension of participant background. Gender identity was categorized such that individuals identifying exclusively as “man” were classified as “cis,” while all others, including transgender women, nonbinary individuals, and other gender identities, were grouped as “gender diverse.” Individual gender identity subgroups were too small to support stable separate estimates. Finally, HIV status was classified as either HIV-positive or HIV-negative.

These standardized categorizations ensured comparability across analyses, improving descriptive statistics and logistic regression modeling while addressing dataset imbalances.

### Data Analysis

#### Overview

All analyses were conducted with Python 3 in Google Colaboratory, using pandas [13], scikit-learn [14], imbalanced-learn [15], and statsmodels [16]. These libraries were selected to support data preprocessing, statistical analysis, and machine learning (ML)–based modeling. The dataset was first summarized using descriptive statistics to provide an overview of participant characteristics and retention patterns.

The analysis was structured into 3 components: primary analysis, secondary analysis, and exploratory analysis, each designed to address its specific objectives.

#### Primary Analysis

The primary analysis examined the association between recruitment source and participant retention. We used Pearson’s chi-squared test and Fisher exact test to assess retention differences across the 4 recruitment sources: social media, partner-seeking apps, study contacts, and other sources. These statistical tests were chosen for their suitability with categorical data and robustness in handling imbalanced sample sizes. Then, odds ratios (OR) and corresponding 95% CIs were calculated. This analysis aimed to determine whether specific recruitment sources were associated with higher participant retention rates.

To evaluate whether the association between recruitment source and retention was independent of demographic differences across sources, a multivariable logistic regression was conducted using statsmodels. The model specified retention (≥3 of 4 follow-up surveys) as the outcome and included recruitment source as the primary exposure variable, with age, HIV status, race, ethnicity, and gender identity included as covariates. Partner-seeking apps served as the reference category for recruitment source, given their representational dominance in the sample (n=394, 52.5%). Reference categories for covariates were as follows: 15 to 19 years (age), HIV-negative (HIV status), non-White or multiracial (race), non-Hispanic (ethnicity), and cisgender man (gender identity). The results are reported as adjusted odds ratios (aOR) with 95% CIs and *P* values. Adjusted pairwise ORs between all recruitment source pairs were obtained by respecifying the model with each category as the reference in turn.

#### Sensitivity Analysis

To assess the robustness of the primary findings to the choice of retention threshold, a sensitivity analysis was conducted using a stricter definition, classifying high retention as the completion of all 4 follow-up surveys. The chi-square test and multivariable logistic regression were repeated using identical model specifications and reference categories as the primary analysis.

#### Secondary Analysis

The secondary analysis explored demographic patterns across the largest recruitment sources, specifically comparing social media and partner-seeking apps. These 2 sources were prioritized because they accounted for the majority of participants, ensuring sufficient statistical power. Recruitment categories with lower representation (ie, study contacts and other sources) were excluded as they would have limited statistical power for meaningful comparisons. Detailed recruitment source distributions are provided in the *Results* section. We used Pearson chi-squared test and Fisher exact test to examine differences in age, ethnicity, race, HIV status, and gender identity distributions between participants recruited through social media compared to partner-seeking apps.

#### Exploratory Analysis

Advances in AI and ML have revolutionized data analysis across disciplines, yet their application in social and behavioral sciences remains relatively underexplored [17]. Traditional inferential models, which emphasize statistical significance and hypothesis testing, dominate behavioral health research [18]. In contrast, predictive models, such as ML-driven logistic regression, focus on identifying patterns in complex datasets and evaluating how well models generalize to new data. By leveraging these strengths, predictive modeling offers an opportunity to enhance the identification of factors influencing participant retention.

To explore predictors of study retention, we developed a predictive logistic regression model incorporating recruitment source, age, ethnicity, race, HIV status, and gender identity as predictors. In this exploratory analysis, the model was used primarily to identify patterns associated with retention rather than to test formal causal hypotheses. We used logistic regression coefficients (β) to assess both the direction and relative magnitude of associations between predictors and retention. Because these coefficients provide interpretable, directional estimates (indicating whether a factor increases or decreases the likelihood of retention), this approach allows for a direct understanding of how specific characteristics influence longitudinal participation.

To develop and evaluate the model, we split the dataset into a training set (n=600, 80%) and a testing set (n=150, 20%) [19]. The training set was used to train the predictive model, while the testing set provided an independent evaluation of model performance on new, unseen data [18]. High-retention participants comprised approximately 80% (598/750) of the sample, creating a class imbalance that could bias models toward predicting retention. To address this, we tested two logistic regression models:

1. Baseline model: Trained on the original, imbalanced dataset.
2. SMOTE-adjusted model: Trained on a dataset where the minority class (low retention) was oversampled using the SMOTE, which generates synthetic samples for the minority class by interpolating between existing data points rather than simply duplicating cases.

Comparing the 2 models allowed us to assess whether balancing the training data improved sensitivity to low-retention participants, and at what cost to overall accuracy, since oversampling may introduce artificial patterns that do not generalize well [20]. The original class distribution was preserved in the testing set throughout.

To systematically compare model performance, we used four standard ML evaluation metrics:

- Precision: The proportion of correctly predicted high or low retention cases out of all cases predicted as high or low retention.
- Recall*:* The proportion of actual high or low retention cases identified by the model.
- *F*_1_-score: A harmonic mean of precision and recall, balancing sensitivity and specificity.
- Accuracy: The overall proportion of correctly classified cases across all retention levels.

By splitting the data into training and testing sets, this analysis ensured that model performance reflected its predictive power rather than overfitting to the dataset [19]. This predictive modeling framework prioritizes generalizability, allowing for more reliable identification of potential retention predictors in future research. Additionally, SMOTE corrects for class imbalances, reducing bias that might otherwise affect standard logistic regression models.

This hypothesis-generating framework complements traditional inferential methods by identifying emerging patterns and potential predictors of retention that may warrant further investigation in future studies.

### Ethical Considerations

The HMP 2.0 RCT was approved by the University of Pennsylvania institutional review board (IRB; protocol #829805) and registered on ClinicalTrials.gov (NCT03678181). All participants provided informed consent prior to their participation, with individuals aged 15 to 17 years granted a waiver of parental consent to protect their privacy as sexual minority youth. To ensure confidentiality, all collected data were deidentified prior to analysis.

## Results

### Participant Characteristics

A total of 750 participants were enrolled in the study between July 2020 and September 2022. The majority (n=445, 59.3%) were aged 25 to 29 years, followed by 236 (31.5%) participants aged 20 to 24 years and 69 (9.2%) participants aged 15 to 19 years. In terms of ethnicity, 318 (42.4%) participants identified as Hispanic, while 432 (57.6%) participants identified as non-Hispanic. Regarding race, 609 (81.2%) participants identified as non-White or multiracial.

Among study participants, 230 (30.7%) reported living with HIV, while 520 (69.3%) were HIV-negative. Most participants (n=666, 88.8%) identified as cisgender men, while 84 (11.2%) participants were categorized as gender diverse, including transgender women (n=16), nonbinary individuals (n=64), and other gender identities (n=4).

Participants were recruited through 4 primary sources. The largest proportion (n=394, 52.5%) were enrolled via partner-seeking apps. Social media platforms accounted for 202 (26.9%) participants, followed by prior study contacts, which contributed 118 participants (15.7%). The remaining 36 participants (4.8%) were recruited through other sources, including peer referrals, in-person outreach, and LGBTQ+ forums. Participant demographics and recruitment sources are summarized in Table 1.

**Table 1.** Demographic characteristics and recruitment sources of young Black and Latino men who have sex with men and transgender women who have sex with men (YBLMT) who participated in HealthMpowerment (HMP) 2.0 (N=750).

Characteristic	Participants, n (%)
Age (y)	
15‐19	69 (9.2)
20‐24	236 (31.5)
25‐29	445 (59.3)
Ethnicity	
Hispanic	318 (42.4)
Non-Hispanic	432 (57.6)
Race	
White	141 (18.8)
Non-White or multiracial	609 (81.2)
HIV status	
HIV-positive	230 (30.7)
HIV-negative	520 (69.3)
Gender identity	
Cisgender man	666 (88.8)
Gender diverse	84 (11.2)
Recruitment source	
Social media	202 (26.9)
Partner-seeking apps	394 (52.5)
Study contacts	118 (15.7)
Other sources	36 (4.8)

Survey completion varied across participants. The majority (n=514, 68.5%) completed all 4 follow-up surveys. An additional 84 (11.2%) participants completed 3 surveys, meeting the high retention threshold. The remaining 152 (20.3%) participants were classified as low retention, including 36 (4.8%) who completed 2 surveys, 36 (4.8%) who completed 1 survey, and 80 (10.7%) who did not complete any follow-up surveys.

### Primary Analysis: Association Between Recruitment Source and Participant Retention

A significant association was observed between recruitment source and participant retention (*χ*²_3_=36.62, N=750; *P*<.001). Retention rates varied across recruitment sources. Retention rates were the highest among participants recruited via social media and study contacts, while those recruited from partner-seeking apps and other sources had lower retention rates.

**Figure 1.**
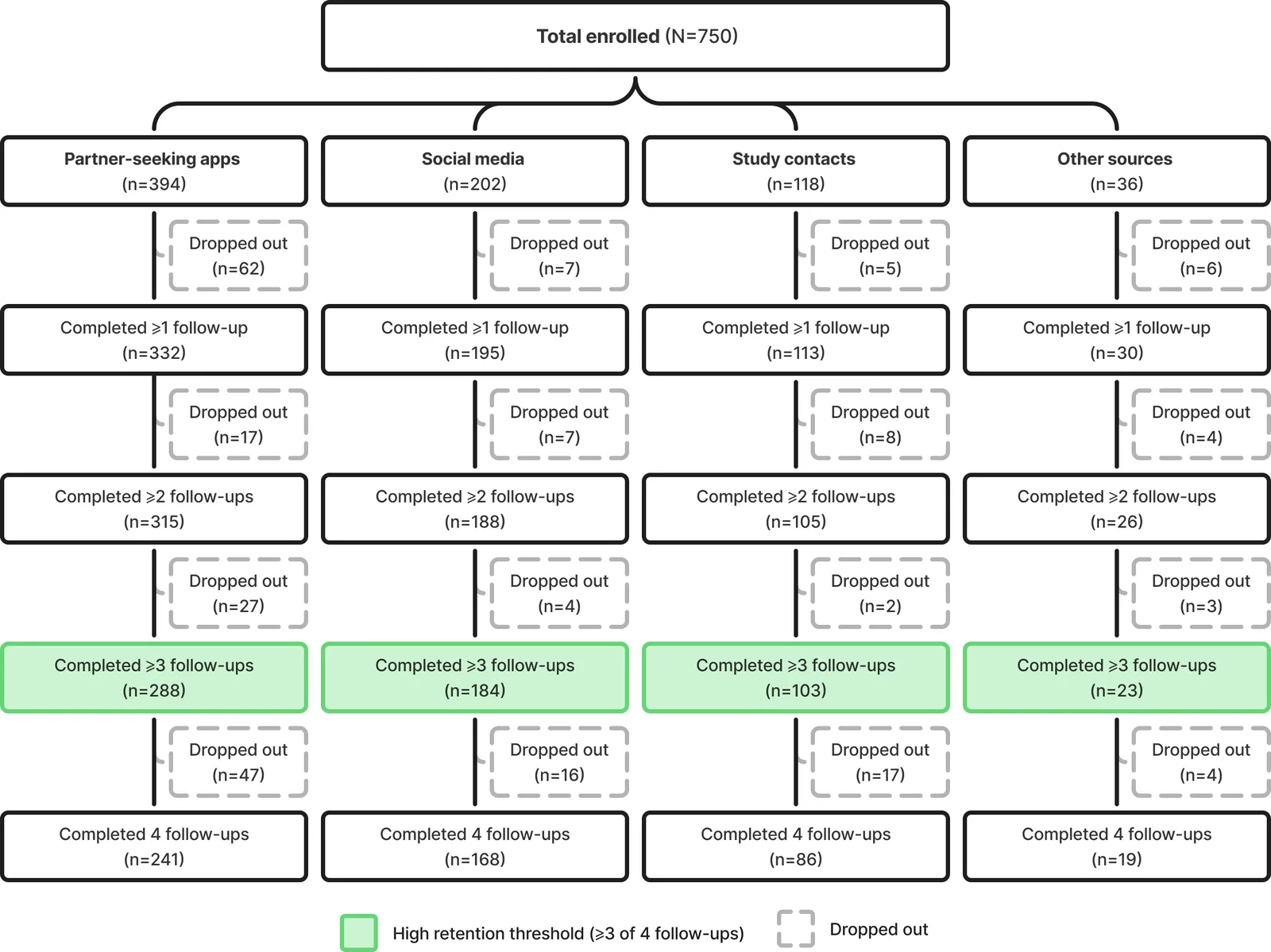
Flow of total study sample stratified by the 4 primary recruitment sources. Participant progression is tracked sequentially across 4 follow-up intervals, detailing the number of participants retained and those who dropped out at each specific stage. The highlighted tier (in green) represents the study’s predefined threshold for high retention.

Among participants recruited via social media, 184 (91.1%) participants met the high retention criterion (completing at least 3 follow-up surveys; Figure 1). Similarly, 103 (87.3%) participants recruited via study contacts were highly retained. In contrast, participants recruited via partner-seeking apps had a retention rate of 288 (73.1%) participants, while those recruited via other sources had a retention rate of 23 (63.9%) participants.

Fisher exact tests revealed significant differences in retention between social media and other recruitment sources (Table 2). Retention was significantly higher among participants recruited via social media compared to those recruited via partner-seeking apps (OR 3.76, 95% CI 2.21‐6.41; *P*<.001) and other sources (OR 5.78, 95% CI 2.51‐13.31; *P*<.001). Retention did not significantly differ between social media and study contacts (OR 1.49, 95% CI 0.72‐3.08; *P*=.34). Participants recruited via study contacts also had significantly higher retention than those recruited via partner-seeking apps (OR 2.53, 95% CI 1.41‐4.54; *P*=.001) and other sources (OR 3.88, 95% CI 1.63‐9.26; *P*=.003). Retention did not significantly differ between partner-seeking apps and other sources (OR 1.54, 95% CI 0.75‐3.14; *P*=.25).

**Table 2.** Retention rates and pairwise comparisons by recruitment source.

Comparison	OR^a^ (95% CI)	*P* value
Social media vs partner-seeking apps	3.76 (2.21-6.41)	<.001
Social media vs other sources	5.78 (2.51-13.31)	<.001
Social media vs study contacts	1.49 (0.72-3.08)	.34
Study contacts vs partner-seeking apps	2.53 (1.41-4.54)	.001
Study contacts vs other sources	3.88 (1.63-9.26)	.003
Partner-seeking apps vs other sources	1.54 (0.75-3.14)	.25

a OR: odds ratio.

To evaluate whether these differences were independent of demographic variation across recruitment sources, a multivariable logistic regression was conducted adjusting for age, HIV status, race, ethnicity, and gender identity (N=750; pseudo *R*²=0.092; Akaike information criterion [AIC]=706.6). After adjustment, participants recruited via social media (aOR 2.80, 95% CI 1.51‐5.18; *P*=.001) and study contacts (aOR 2.11, 95% CI 1.14‐3.92; *P*=.02) remained significantly associated with higher odds of retention compared to those recruited via partner-seeking apps. Other sources did not differ significantly from partner-seeking apps after adjustment (aOR 0.61, 95% CI 0.28‐1.31; *P*=.21). Full model results are presented in Table 3.

**Table 3.** Multivariable logistic regression: adjusted association between recruitment source and retention (≥3 of 4 surveys; N=750; model fit: pseudo *R*²=0.092; AIC=706.6).

Predictor	aOR^a^ (95% CI)	*P* value
Recruitment source (reference: partner-seeking apps)		
Social media	2.80 (1.51-5.18)	.001
Study contacts	2.11 (1.14-3.92)	.018
Other sources	0.61 (0.28-1.31)	.21
Age (y; reference: 15‐19)		
20‐24	0.48 (0.21-1.08)	.08
25‐29	0.95 (0.42-2.13)	.90
HIV status (reference: HIV-negative)		
HIV-positive	0.64 (0.42-0.96)	.03
Race (reference: Non-White or multiracial)		
White	1.22 (0.60-2.45)	.59
Ethnicity (reference: Non-Hispanic)		
Hispanic	1.20 (0.74-1.95)	.45
Gender identity (reference: cisgender man)		
Gender diverse	0.38 (0.23-0.63)	<.001

a aOR: adjusted odds ratio.

Among covariates, HIV-positive status was independently associated with lower odds of retention (aOR 0.64, 95% CI 0.42‐0.96; *P*=.03). Gender-diverse participants also had significantly lower odds of retention compared to cisgender men (aOR 0.38, 95% CI 0.23‐0.63; *P*<.001). Age, race, and ethnicity were not independently associated with retention after adjustment.

Adjusted pairwise comparisons across all 4 recruitment source pairs are presented in Table 4. Other sources had significantly lower adjusted odds of retention compared to both social media (aOR 0.22, 95% CI 0.09‐0.54; *P*=.001) and study contacts (aOR 0.29, 95% CI 0.12‐0.72; *P*=.007). Social media and study contacts did not differ significantly from each other after adjustment (aOR 0.76, 95% CI 0.35‐1.61; *P*=.47).

**Table 4.** Adjusted pairwise odds ratios for retention (≥3 of 4 surveys) across all recruitment source pairs, adjusted for age, HIV status, race, ethnicity, and gender identity.

Comparison	aOR^a^ (95% CI)	*P* value
Social media vs partner-seeking apps	2.80 (1.51-5.18)	.001
Study contacts vs partner-seeking apps	2.11 (1.14-3.92)	.02
Other sources vs partner-seeking apps	0.61 (0.28-1.31)	.21
Study contacts vs social media	0.76 (0.35-1.61)	.47
Other sources vs social media	0.22 (0.09-0.54)	.001
Other sources vs study contacts	0.29 (0.12-0.72)	.007

a aOR: adjusted odds ratio.

### Sensitivity Analysis

Using a strict definition (completion of all 4 surveys; n=514, 68.5% of the participants), retention rates were 83.2% (168/202) for social media, 72.9% (86/118) for study contacts, 61.2% (241/394) for partner-seeking apps, and 52.8% (19/36) for other sources. The overall *χ*^2^ remained significant (*χ*²_3_=35.15, N=750; *P*<.001). Social media remained significantly associated with higher odds of retention compared to partner-seeking apps (aOR 2.79, 95% CI 1.70‐4.57; *P*<.001). The advantage for study contacts was attenuated and no longer reached significance (aOR 1.58, 95% CI 0.97‐2.55; *P*=.07).

### Secondary Analysis: Demographic Patterns Across the Two Largest Recruitment Sources

#### Overview

Demographic characteristics varied significantly between participants recruited via social media and those recruited via partner-seeking apps. Analyses were limited to these 2 sources (n=596), as they accounted for the majority of participants. Demographic patterns across the 2 largest recruitment sources are summarized in Table 5.

**Table 5.** Demographic differences between participants recruited via social media and partner-seeking apps (n=596).

Characteristic	Social media (n=202), n (%)	Partner-seeking apps (n=394), n (%)	Chi-square (*df*)	*P* value
Age (y)			33.88 (2)	<.001
15‐19	29 (14.4)	11 (2.8)		
20‐24	73 (36.1)	122 (31.0)		
25‐29	100 (49.5)	261 (66.2)		
Race			116.27 (1)	<.001
White	82 (40.6)	20 (5.1)		
Non-White or multiracial	120 (59.4)	374 (94.9)		
Ethnicity			152.56 (1)	<.001
Hispanic	153 (75.7)	90 (22.8)		
Non-Hispanic	49 (24.3)	304 (77.2)		
HIV status			88.91 (1)	<.001
HIV-positive	11 (5.4)	171 (43.4)		
HIV-negative	191 (94.6)	223 (56.6)		
Gender identity			.77 (1)	.38
Cisgender man	183 (90.6)	346 (87.8)		
Gender diverse	19 (9.4)	48 (12.2)		

#### Age

Participants recruited via social media (n=202) were younger on average, including those aged 15 to 19 years (n=29, 14.4%), 20 to 24 years (n=73, 36.1%), and 25 to 29 years (n=100, 49.5%). By contrast, participants recruited through partner-seeking apps (n=394) were generally older, including those aged 15 to 19 years (n=11, 2.8%), 20 to 24 years (n=122, 31.0%), and 25 to 29 years (n=261, 66.2%). A chi-square test confirmed significant differences in age distribution between the 2 groups (*χ*²_2_=33.88, n=596; *P*<.001).

#### Race or Ethnicity

A higher proportion of participants recruited via social media identified as White (82/202, 40.6%), whereas a higher proportion of those recruited via partner-seeking apps identified as non-White or multiracial (374/394, 94.9%). Similarly, social media recruitment yielded a predominantly Hispanic participant pool (153/202, 75.7%), whereas participants recruited from partner-seeking apps were less likely to identify as Hispanic (90/394, 22.8%). Chi-squared tests confirmed significant differences in both race (*χ*²_1_=116.27, n=596; *P*<.001) and ethnicity (*χ*²_1_=152.56, n=596; *P*<.001) distributions between the 2 recruitment sources.

#### HIV Status

HIV-negative participants accounted for the vast majority (191/202, 94.6%) of those recruited via social media, whereas a higher proportion (171/394, 43.4%) of the participants recruited via partner-seeking apps were HIV-positive. Chi-square test results confirmed that HIV status distributions differed significantly between recruitment sources (*χ*²_1_=88.91, n=596; *P*<.001).

#### Gender Identity

The distribution of gender identity was largely similar across both recruitment sources, with cisgender men comprising 90.6% (183/202) of participants recruited through social media and 87.8% (346/394) of those recruited through partner-seeking apps. The chi-square test found no significant difference in gender distribution between the 2 recruitment sources (*χ*²_1_=0.77, n=596; *P*=.38).

### Exploratory Analysis: Predictors of Participant Retention

The baseline model achieved an accuracy of 77.3%. It correctly classified 97.4% (114/117) of the participants with high retention but only 6.1% (2/33) of the participants with low retention. The resulting *F*_1_-score for low-retention participants was 0.11, reflecting limited precision and recall for this group.

To improve sensitivity in classifying participants with low retention, the SMOTE-adjusted model was trained on a dataset with an artificially balanced distribution of retention groups. This model achieved a lower overall accuracy of 64.7%. However, recall for low-retention participants increased from 6.1% (2/33) to 63.6% (21/33), and the *F*_1_-score for this group improved from 0.11 to 0.44. Recall for high-retention participants decreased from 97.4% (114/117) to 65.0% (76/117). Full performance metrics for both models are presented in Table 6.

**Table 6.** Comparison of model performance.

Model	Accuracy, n/N (%)	Precision (low retention), n/N (%)	Recall (low retention), n/N (%)	*F*_1_-score (low retention)	Precision (high retention), n/N (%)	Recall (high retention), n/N (%)	*F*_1_-score (high retention)
Baseline	116/150 (77.3)	2/5 (40.0)	2/33 (6.1)	0.11	114/145 (78.6)	114/117 (97.4)	0.87
SMOTE^a^-adjusted	97/150 (64.7)	21/62 (33.9)	21/33 (63.6)	0.44	76/88 (86.4)	76/117 (65.0)	0.74

a SMOTE: Synthetic Minority Oversampling Technique.

Beyond overall model performance, logistic regression coefficients were examined to assess the relative contribution of each predictor relative to its reference group (Table 7). These coefficients represent the change in the log-odds of high retention when moving from the reference category to the comparison category for each predictor variable, holding all other variables constant. Because all predictors in the model are categorical, their coefficients should be interpreted as the relative likelihood of high retention compared to the reference group. Recruitment source remained the strongest predictor, with social media (β=1.45) and study contacts (β=1.29) showing the largest positive associations with retention compared to partner-seeking apps. These findings were directionally consistent with the adjusted inferential analysis. HIV-positive status (β=−.37) and gender-diverse identity (β=−0.75) showed negative associations with retention, also consistent with the inferential model. The coefficient for other sources (*β*=0.74) was directionally inconsistent with the adjusted regression, likely reflecting instability in the small subsample available for model training (n≈29 after the 80/20 split); the adjusted logistic regression is the more reliable estimate for that comparison.

**Table 7.** Synthetic Minority Oversampling Technique (SMOTE)–adjusted logistic regression coefficients for predictors of participant retention (≥3 of 4 surveys).

Comparison	Coefficient
Social media vs partner-seeking apps	1.45
Study contacts vs partner-seeking apps	1.29
White vs non-White or multiracial	0.82
Other sources vs partner-seeking apps	0.74
Age 25‐29 vs age 15‐19	0.54
Hispanic vs non-Hispanic	0.07
Age 20‐24 vs age 15‐19	0.00
HIV-positive vs HIV-negative	–0.37
Gender diverse vs cisgender man	–0.75

## Discussion

### Principal Findings

Our findings demonstrate that recruitment source is associated with participant retention in online health interventions for YBLMT. By evaluating how different platforms correspond to retention outcomes, we highlight the need for strategic recruitment planning that goes beyond sample diversity to consider sustained participation over time.

In line with prior research [21,22], our primary analysis found that retention rates differed significantly across recruitment sources. Participants recruited via social media and prior study contacts maintained the highest retention (184/202, 91.1% and, 103/118, 87.3%, respectively). This may reflect platform norms: social media users often engage in longitudinal interactions (such as browsing, commenting, and following content over time), which may translate more effectively to long-term study participation [21]. The higher retention observed among prior study contacts likely reflects preexisting institutional trust and an established rapport with the research team [23,24]. Individuals who have previously participated in research and opted into a recontact listserv may possess a higher baseline motivation and a more favorable view of the research process, significantly increasing their likelihood of completing longitudinal follow-up.

In contrast, the lower retention (288/394, 73.1%) observed among participants recruited via partner-seeking apps may reflect the “low-friction” and highly goal-oriented nature of those platforms that may not align with sustained engagement [25]. Users may engage with a recruitment ad out of momentary curiosity or during a brief window of app usage without the baseline commitment or institutional trust found in social media networks or prior research listservs. This suggests that while apps are excellent for reaching high-risk populations, they may require more intensive “onboarding” to bridge the gap between initial click and long-term retention. Community-based recruitment (comprising in-person outreach, peer referrals, and LGBTQ+ forum postings) was associated with the lowest adjusted retention across all 4 sources, which may reflect greater structural or logistical barriers to sustained digital study participation among community-recruited participants and warrants further investigation.

Retention differences may also reflect the distinct participant characteristics associated with each recruitment source, as demonstrated in our secondary analysis. Social media recruitment yielded a younger, predominantly Hispanic, and HIV-negative cohort, whereas partner-seeking apps recruited older, non-White, and HIV-positive participants. Although retention rates were lower among participants recruited via partner-seeking apps, continued recruitment through these platforms is warranted, given that they reach large populations living with or vulnerable to HIV. Allocating additional resources for follow-up efforts, such as tailoring reminder strategies to participants’ recruitment platforms, may support retention in these groups. By contrast, the high retention observed among prior study contacts suggests that formalized participant referral programs are a useful strategy for sustaining retention in future studies. These patterns highlight the intersectional nature of recruitment dynamics and the importance of adjusting for potential confounders when estimating the independent association between recruitment source and retention.

Participants recruited via partner-seeking apps represented a cohort that was significantly older, more racially diverse, and more likely to be living with HIV compared to those from social media. These overlapping identities and statuses may correlate with unique structural demands (such as navigating health care systems for HIV care or differing digital engagement habits) that likely influenced their lower retention rates.

After adjusting for age, HIV status, race, ethnicity, and gender identity, social media and study contacts remained significantly associated with higher retention compared to partner-seeking apps, indicating that the retention advantage observed in these channels is not fully explained by the demographic differences in who they recruit. Beyond recruitment source, 2 demographic factors were independently associated with lower retention after adjustment. HIV-positive participants had significantly lower adjusted odds of retention (aOR 0.64), consistent with evidence that individuals managing HIV face competing health demands, stigma-related burdens, and structural barriers that may limit sustained engagement with digital health programs [1]. Gender-diverse participants had substantially lower adjusted odds of retention (aOR 0.38), a finding that persisted across the full multivariable model. Transgender and gender-nonconforming individuals experience elevated rates of housing instability, employment discrimination, and mistrust of health and research institutions [1], any of which may affect their ability to maintain participation over a 12-month study period. These findings suggest that retention support efforts should not only be tailored to recruitment platforms but also prioritize outreach to HIV-positive and gender-diverse participants, who face independently elevated risks of dropout regardless of how they were enrolled.

A sensitivity analysis using a stricter retention definition (ie, completion of all 4 follow-up surveys) was directionally consistent with the primary findings. The social media advantage over partner-seeking apps remained significant under this stricter threshold, confirming that this association is not an artifact of the chosen retention cutoff. The study contacts advantage attenuated and no longer reached significance when full completion was required, suggesting that prior study participants reengage readily but may face the same real-world barriers to sustained long-term participation as other groups. This distinction is worth considering in future studies that rely heavily on prior participant networks for recruitment.

To complement the inferential analysis, we developed a predictive logistic regression model to explore whether demographic and recruitment variables could classify retention outcomes—a hypothesis-generating exercise motivated by the potential of ML approaches to surface patterns in behavioral health data [17,18]. The baseline model achieved high accuracy overall (77.3%) but classified only 6.1% (2/33) of low-retention participants correctly, reflecting the limitations of standard logistic regression under class imbalance. Because the dataset was heavily skewed toward high-retention participants, the model’s estimates for predictors of disengagement were unreliable without correction.

To address this, we applied SMOTE to synthetically balance the training dataset. The SMOTE-adjusted model achieved an accuracy of 64.7%, with low-retention recall improving from 6.1% (2/33) to 63.6% (21/33) and the *F*_1_-score for that group improving from 0.11 to 0.44, at the cost of a reduction in overall accuracy. Importantly, the goal of applying SMOTE was not to predict which individual participants would drop out, but to strengthen the model’s ability to detect group-level patterns associated with lower retention. The direction of the SMOTE model coefficients was largely consistent with the adjusted inferential analysis: social media and study contacts showed the strongest positive associations with retention, while HIV-positive status and gender-diverse identity were negatively associated. The coefficient for community-based recruitment diverged from the adjusted regression estimate, likely reflecting instability given the small subsample available for model training (n≈29 after the 80/20 split); the adjusted logistic regression provides the more reliable estimate for that comparison.

Our findings suggest that retention strategies should be tailored based on the recruitment platform’s digital “friction.” For participants recruited through highly goal-oriented channels such as partner-seeking apps, informatics-based interventions could incorporate targeted engagement features to mimic the rapport seen in established research networks. Specific digital “nudges” might include personalized push notifications tailored to the participant’s study milestone, milestone-based incentives, and gamified progress indicators that visually reward longitudinal participation. Additionally, incorporating peer interaction prompts or “community boards” may help build the institutional trust and social connectivity that characterized our high-retention study contact group. These design strategies offer a scalable way to optimize both participant diversity and sustained study engagement.

### Limitations and Future Directions

This study has several limitations. First, the race variable was collected using a multiselect item, which precluded disaggregation beyond a White versus non-White or multiracial binary classification. Given that the study specifically targets Black and Latino YBLMT, future analyses should use survey instruments that capture racial subgroup membership in a format amenable to group-level comparisons, enabling more granular examination of retention differences within the non-White population.

Although age was not independently associated with retention in the primary adjusted regression, the youngest cohort (15‐19 y, n=69) showed relatively high apparent retention, and we recognize that age may nevertheless serve as a proxy for competing life responsibilities. Factors such as full-time employment, independent housing logistics, and other adult responsibilities (which correlate with older age in this demographic) may serve as barriers to longitudinal engagement compared to younger participants who may have more discretionary time. Since these specific socioeconomic status indicators were not collected in the parent trial, we cannot definitively separate the impact of life stage from economic stability. Future research should prioritize the inclusion of comprehensive socioeconomic status metrics to better understand these overlapping influences on study persistence. Additionally, reliance on self-reported data and survey completion introduces the possibility of selection or response bias. Beyond these concerns, our study did not assess individual-level factors such as motivation, competing life responsibilities, or platform usability. Integrating qualitative data or engagement analytics could provide deeper insights into these factors.

Because this analysis was conducted using data from a parent RCT, the study was not originally powered to detect differences in retention across recruitment sources. The findings should therefore be interpreted as exploratory and hypothesis-generating. Although multiple digital platforms were used for recruitment (eg, Facebook, Instagram, Grindr, Jack’d, Scruff), the sample size within individual platforms was insufficient to conduct reliable platform-specific comparisons. Future research examining cross-platform comparisons may be warranted [26], as it may elucidate how differences in platform design, user demographics, and interaction norms influence both recruitment efficiency and longitudinal engagement in digital health interventions. Such analyses could help researchers more strategically allocate recruitment resources and tailor retention strategies to the behavioral patterns and expectations associated with specific digital environments.

Our logistic regression model successfully identified key predictors of retention, including recruitment source, age, race, gender identity, and HIV status. However, its ability to classify low-retention participants was limited, pointing to the need for refinement of predictive approaches. A sensitivity analysis using a stricter retention definition (completion of all 4 surveys) was conducted and found to be directionally consistent with the primary findings, though the study contacts advantage attenuated under this threshold.

Finally, we used SMOTE to address class imbalance in retention outcomes. While this method improved recall for low-retention participants from 6.1% (2/33) to 63.6% (21/33) and increased the *F*_1_-score from 0.11 to 0.44, it reduced overall accuracy from 77.3% to 64.7%. Other resampling or model-weighting techniques may yield different trade-offs and should be explored in future work.

### Conclusions

Recruitment source was associated with participant retention in this online health intervention, highlighting its importance in digital research planning. Some platforms correspond to higher retention, while others enhance sample diversity or reach populations at increased HIV risk. As digital advertising costs rise, researchers must weigh recruitment efficiency against retention feasibility. By refining recruitment strategies and retention frameworks, future online HIV interventions can be optimized to better sustain participation among populations most impacted by the epidemic.

## Abbreviations

AIC: Akaike Information Criterion
aOR: adjusted odds ratio
CASI: computer-assisted self-interview
HMP: HealthMpowerment
IRB: institutional review board
ML: machine learning
OR: odds ratio
RCT: randomized controlled trial
SMOTE: synthetic minority oversampling technique
USPSTF: United States Preventive Services Task Force
YBLMT: Young Black and Latino men who have sex with men and transgender women who have sex with men
